# Improving Recovery of Diatoms Bio-Silica Using Chemical Treatment with VAUS TM

**DOI:** 10.21203/rs.3.rs-4709095/v1

**Published:** 2024-08-02

**Authors:** Se Ryung Suh, Joo Hun Lee, Gyung Min Go, Jaeyoung Lee, Hyunjoon Kong, Eun-Jin Park

**Affiliations:** Ewha Womans University; University of Illinois; JDK Bio Inc; Mirae Ultrasonic Tech; University of Illinois; Ewha Womans University

**Keywords:** diatom, bio-silica, vacuum, ultrasonic, organic matter, M. nummuloides

## Abstract

High-temperature baking is a typical method to remove organic matter from diatoms, but it’s not suitable for bio-silica because of the high crystallinity. This study provides a method using the VAUS^™^ to remove organic matter from diatoms more quickly and biocompatibly. Organic matter was removed by using VAUS^™^, while NaOCl was utilized to remove the organic matter from the silicate frustule. The optimal frequency for organic matter removal was investigated to domestically produced *M. nummuloides.* The removal efficiency of TOC/TN was calculated and analyzed. The C and Si elements were analyzed in EDS, while visual confirmation of organic matter removal was analyzed by using XRD. TOC RE% at a frequency of 35kHz exhibited the highest value, indicating a statistically significant difference. XRD analysis demonstrated that the organic matter was almost removed using NaOCl compared to the high-temperature baked *M. nummuloides*. In the EDS analysis, there were significant differences in the C and Si elements with respect to frequency. This is very similar to the values from the positive control group, high-temperature baked *M. nummuloides.* This new procedure of applying periodic negative pressure to NaOCl pre-treatment is considered to be an effective method of chemically removing organic matter from diatoms.

## Introduction

Recent societal trends show a rapid shift towards an aging population, with the elderly now constituting over 20% of the overall population. Accompanying this demographic transition is a noticeable surge in dental prosthetic users. Research involving Dental Health Care Professionals (DHCPs) from various nations and patients using dental prosthetics revealed that 1 in 7 DHCPs did not recommend primary methods due to a lack of evidence-based consensus. On the other hand, dental prosthetic users employed various cleansing methods, including saline, bleach, vinegar, and baking soda.^1^

Biofilms are sessile microbial communities growing on surfaces, frequently embedded in a matrix of extracellular polymeric substances.^2,3^ However, the surface of the acrylic resins can be colonized by a microbial biofilm, causing lesions of the buccal mucosa and favoring the development of systemic infections.^4–6^ It is extremely important that chemical denture cleansers be used as an adjunct for patients unable to properly care for their dentures and manage overall oral hygiene.^7^ Although Dental Cleaning Agents (DCA) are effective in removing mild tartar or slight discoloration, mechanical cleansing method remains essential and recommended due to the tenacious long-term adhesion of biofilm.^8–10^

In this study, *Melosira nummuloides*, lava seawater diatoms primarily found during the low-temperature winter periods in the Northern Hemisphere’s temperate regions, were identified in certain areas of Jeju Island. These diatoms effectively block heavy metals and harmful substances, leading to reduced chances of contamination. Moreover, the lava seawater maintains consistent temperature and salinity levels throughout the year, negating the need for additional installations such as filtration, sterilization, or heating units for diatom processing. Notably, the procurement cost of lava seawater is 25–75 times less expensive compared to deep ocean water, making it a viable option for culturing purposes.

Consequently, *M. nummuloides* was used as a negative control group in this study to compare organic matter removal rates under economically feasible mass culture conditions. Furthermore, the study aimed to explore the potential applications of constituents found in the mass-cultured *M. nummuloides*. By comparing properties based on different processing methods, it seeks ways to utilize them as high-value-added materials.^11^

Bio-silica derived from *M. nummuloides* boasts a nano-porous structure, specifically a frustule, which provides an expansive surface area. Its resilience to strong acids and high temperatures renders it invaluable for diverse industries, including use as a photocatalyst, adsorbing material, template for nano-catalyst production, sensors, and biocompatible material for biofilm removal.

However, a significant challenge arises in the initial step of producing industrially viable bio-silica, which involves the removal of organic matter.^12,13^ This process often demands the excessive use of hazardous reagents, involves intricate procedures, and requires extended processing times. Moreover, during the extraction process, there is a notable risk of damaging the nano-porous structure. Bio-silica is currently extracted by treating *M. nummuloides* with 10% hydrochloric acid followed by high-temperature baking at 650℃ for 5 hours. However, this method presents several challenges: it involves the use of high concentrations of acid, potentially increases cytotoxicity due to enhanced crystallinity from high-temperature baking, raises environmental pollution concerns, and necessitates an extended duration of the process. In dentistry, essential bone graft materials used for procedures like implant installation face similar challenges. Conventionally, xenogenic bones such as bovine are treated with strong alkalis and subjected to heat treatment at temperatures from 300℃ to 1000℃ for at least 15 hours, followed by gamma-ray sterilization. However, these methods can lead to issues with immune reactions and the osteogenic potential of the grafts. In response, techniques that utilize autogenous teeth as an alternative have been developed.^14^ Inspired by this, our study aims to use periodic negative pressure and ultrasonication instead of high-temperature baking to remove organic matter from diatoms. The current limitation to experimental use in high-value applications, such as in medicine or optics, restricts its industrial application to less lucrative areas, like water purification filters. Our study introduces an eco-friendly method for the removal of organic matter from diatoms using NaOCl, which has the potential to revolutionize industry practices.^15^ Our innovative approach combines the use of a vacuum ultrasonic device capable of periodically applying negative pressure to rapidly and biocompatibly produce bio-silica for diverse industrial applications. We aim to expedite the production process of bio-silica within this research’s scope and mitigate the potential release of toxic substances associated with traditional combustion methods.^16,17^ Notably, by evaluating organic matter removal rates at four distinct frequencies during the prototype phase, and adjusting vacuum and stirring durations, we will determine the optimal conditions for various applications. Such insights will undoubtedly enhance the material’s future applicability. The compact, low-noise design of our base unit allows for installation in confined spaces, facilitating its use in individual research endeavors. Furthermore, the reduced processing time presents a safe and efficient alternative for large-scale production, heralding a future where our breakthrough is widely adopted.

The aim of this study is to address the prevalent use of high-temperature baking for the removal of organic matter from diatoms. While this is a conventional method, it is not ideal for the formation of bio-silica due to its propensity to induce high crystallinity, compromising the material’s structural integrity. To overcome this limitation, we intend to introduce a more rapid and eco-friendly technique employing VAUS^™^, a novel approach aimed at preserving structural integrity of bio-silica. This research seeks to pave the way for an innovative method that ensures optimal bio-silica production without compromising its structural integrity.

## Methods

**Figure 1:**
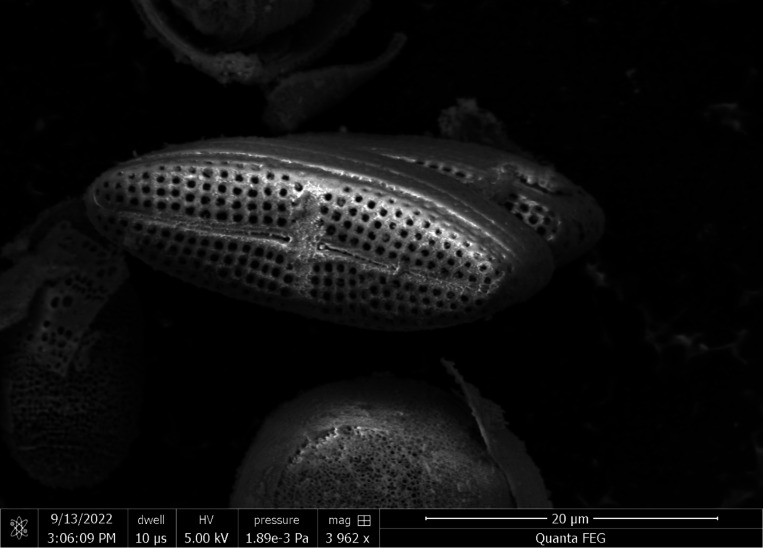
*Melosira nummuloids* from the sea of Korea (JDK Bio Co., Jeju, Korea).

The experimental raw-material, *M. nummuloides* (RM), and the positive control group, bio-silica were provided by JDK Bio Co. (Jeju, South Korea) (Fig. 1). RM was produced from marine diatoms, *M. nummuloides*, isolated from lava seawater at concentrations of 2–4 cells/L. For large-scale cultivation, lava seawater was collected into a pond where *M. nummuloides* was cultured and harvested under optimal conditions. The harvested material was then washed with fresh water to desalinate and remove impurities, followed by natural dehydration to a moisture content of 85% or less through a 100μm mesh filter.

Bio-silica was produced from RM using methods based on freeze-dried *M. nummuloides* (FM) and ethanol-extracted *M. nummuloides* (EM). The process involved drying the diatoms at temperatures between 60–80℃ to remove moisture, followed by grinding. To eliminate organic matter, the material was treated with a hot solution of sulfuric acid/hydrogen peroxide or hydrochloric acid/hydrogen peroxide. Impurities were then removed by treating it with a 10% hydrochloric acid solution at 95℃. After drying at 80℃, the material was washed with distilled water at 650℃, then vacuum dried at temperatures between 100–200℃. The final product was diatom-derived bio-silica, with organic matter removed, ready for collection.

**Figure 2:**
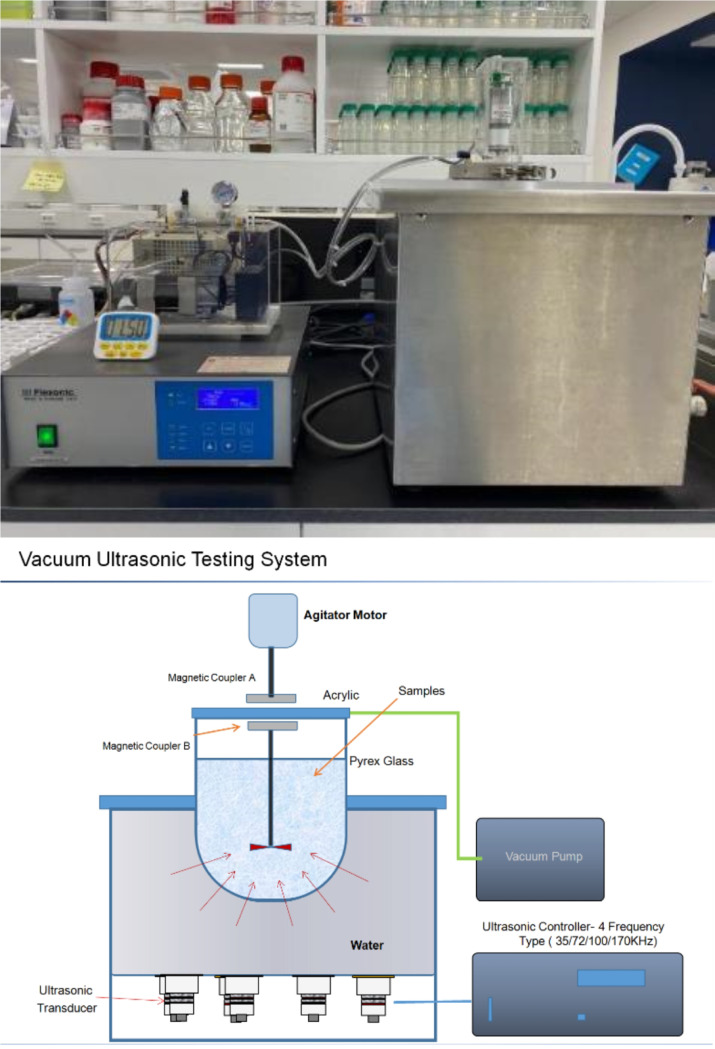
Vacuum Assisted Ultrasonic Stirrer (VAUS^™^, Mirae Ultrasonic Tech., Korea)

The Vacuum Assisted Ultrasonic Stirrer (VAUS^™^, Mirae Ultrasonic Tech., Korea) (Fig. 2) utilized in this study can generate ultrasonic waves ranging from 35–170 kHz, in single or multiple frequencies. A magnetic stirrer incorporated within the vacuum ultrasonic device enables the processing of lightweight particulate materials, potentially surpassing traditional equipment. This feature allows for both intensive and precision cleaning, ensuring a uniform distribution of energy across the entire sample and steady facilitation of reactions. The multi-frequency ultrasonic generator within the device delivers even energy throughout the sample. It also reduces the cavitation size from the ultrasonic vibrator, which assures that the ultrasonic energy effectively penetrates the nano-porous structures, promoting simultaneous internal and external reactions. Negative pressure applied alongside the periodic creation of a vacuum by the vacuum pump swiftly removes simple bubbles, reducing cavitation blind spots and particle acceleration, thereby minimizing energy loss and enhancing the reaction process. The system also includes a magnetic stirrer that maintains fine particulate samples in suspension within the reaction solution, facilitating consistent and expedited cleaning and reactions.

In this study, 4.5% NaOCl was used to remove organic matter from the negative control group RM, instead of the conventional high-temperature baking or chemical solutions. After 50g of RM was mixed with 500ml of 4.5% NaOCl, the mixture was placed in the VAUS^™^ device, which was stirred to prevent solidification. The settings of the VAUS^™^ were adjusted based on frequency, vaccum application, and stirring time. Prolonged stirring increased the temperature within the VAUS^™^ tank, necessitating the addition of ice water to maintain a temperature below 40℃. The mixture was then divided into twelve 50ml conical tubes, each containing 42ml, and the contents were weighed to ensure even distribution. These tubes were centrifuged in a Combi 514R(Hanil, Korea) at 4000rpm for 15 minutes, and the supernatant(4.5% NaOCl) was discarded. The tubes were then paired, reducing the count to six and equalizing the volume to 45ml with the addtion of DI water. This was followed by a 10-minute vacuum ultrasonic cleansing session (Flexonic, Mirae Ultrasonic Tech., Korea). Subsequent to cleansing, the six conical tubes were centrifuged again under the same condition, and the supernatant(DI water) was removed. The remaining tubes were combined into pairs to form three tubes, equalized to 45ml with DI water, and manually shaken for further cleansing. After a final centrifugation at 4000rpm for 15 minutes, the supernatant(DI water) was discarded, and the tubes were stored at a temperature of 10 to 15℃.

The centrifuged samples were rapidly frozen using a freeze dryer (Bondiro, Ilsin lab, Korea) and, with the assistance of a vacuum pump, moisture and other components were removed over 48 hours at temperature typically lower than 10℃, achieving freeze-drying. Equipped with a high-capacity sealed freezing system, the chamber’s interior was quickly cooled to below − 40℃. The samples were dried using a method that involves rolling the flasks, which completely eliminated any chances of effervescence and melting. This method promoted a film-like preliminary freeze, suppressing the surface hardening and concentrated solidification of the samples during freezing, resulting in an optimal drying outcome. After freeze-drying, the samples were sealed with parafilm to prevent moisture and foreign substances from entering. Subsequent analyses and measurements for TOC/TN, EDS, and XRD data were conducted on these samples.

SEM images were captured using Field-Emission Scanning Electronic Microscopy(AURIGA, Carl Zeiss, Germany) to observe the porous structure, particle size, and microstructure of *M. nummuloides* and the sample after organic matter removal.

All samples, including the negative and positive control groups, were analyzed for Total Organic Carbon(TOC) and Total Nitrogen(TN) using the TOC Analyzer(Sievers 5310 C, GE, USA). In the TOC analysis, samples were processed in the UV oxidation chamber, where organic matter was fully oxidized to carbon dioxide(CO_2_). The CO_2_ produced was then captured through a selective membrane, and its quantity was measured using a conductivity detector. In the TN analysis, inorganic carbon dissolved in the sample was completely converted to CO_2_ under acidic conditions. Only the CO_2_ that passed through the selective membrane was captured, and the total nitrogen amount was measured using the conductivity detector. The microprocessor determined the exact amount of TOC by calculating the difference between the total carbon and the inorganic carbon. The analyzed TOC and TN values are denoted as TOCi and TOCf for initial(negative control group) and final(sample) TOC, and TNi and TNf for initial(negative control group) and final(sample) TN values, respectively. These values were then used in an equation to compute the TOC and TN removal efficiency(RE%).

XRD analysis was performed using Powder X-Ray Diffractometry(D8 Advance, Bruker, Germany). Cooper K-alpha radiation was directed at the sample, and the resulting diffraction patterns facilitated the structural analysis of crystalline and amorphous materials, including phase analysis and crystal orientation. In this study, XRD was employed to elucidate the characteristics and structural attributes of frustules before and after the NaOCl process. To visually evaluate the efficiency of organic matter removal, analyses of the crystalline structures of the untreated frustule(UF) and the treated frustule(TF) were conducted.

EDS analysis were conducted using the Field-Emission Scanning Electronic Microscopy (AURIGA, Carl Zeiss, Germany). Measurements were based on the characteristics of secondary electrons and backscattered electrons released after the electron beam interacted with the sample, forming a three-dimensional image. The weight percent(wt.%) of the carbon(C) element was compared to gauge the amount of organic matter before and after ultrasonic treatment. Following NaOCl pre-treatment, the organic matter was removed. Through EDX analysis, it was possible to compare the weight and ratio changes of the C and Si elements during the NaOCl pre-treatment.

All statistical analyses were performed using SPSS for Window(SPSS version 29; IBM Corporation, Armonk, NY, USA). The data’s distribution was evaluated for normality using Kolmogorov-Smirnov and Shapiro-Wilk tests. To compare the means of each experimental group and the control group in the TOC/TN and EDS analyses, t-tests and one-way ANOVA followed by Scheffe’s post hoc tests were performed. P-values less than 0.05 were considered to reflect statistically significant differences.

**Figure 3:**
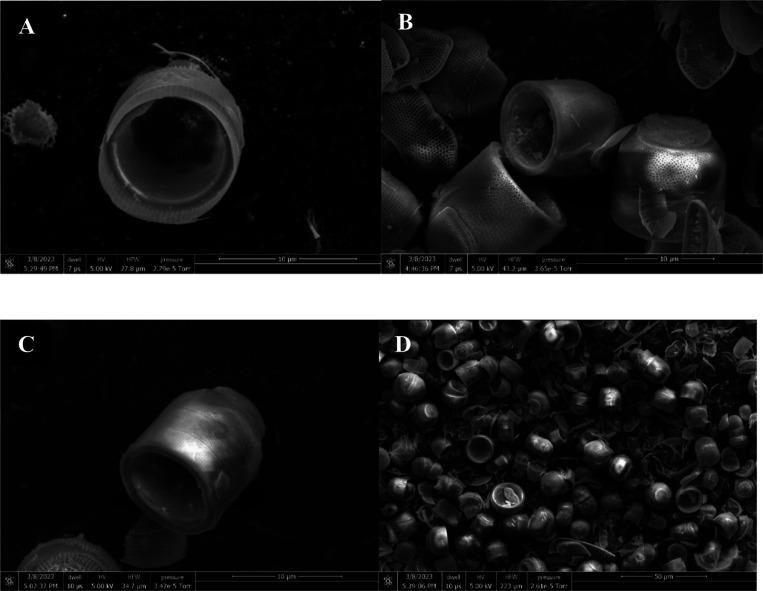
SEM images of *M. nummuloides* after organic matter removal at different frequencies A. 35kHz B. 72kHz C. 100kHz D. 170kHz

SEM images of *M. nummuloides* were captured after the removal of organic matter at different frequencies. Overall, they presented a tendency for some parts to detach, yet no distinct differences were observed between the groups. The average diameter of these particles was measured to be 100–500nm (Fig. 3).

Total Organic Carbon(TOC) analysis did not reveal significant differences based on vacuum application or stirring time. However, at 35kHz, it exhibited the highest value of 83.419 ± 3.052, with a p-value of 0.008, indicating a statistically significant difference. For Total Nitrogen(TN), no significant differences were found with respect to vacuum application, stirring time, or frequency, as all p-values were above 0.05 (Table 1).

**Figure 4:**
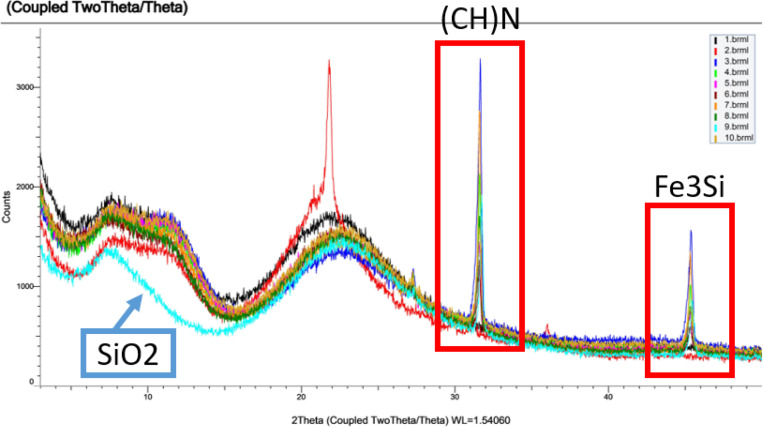
XRD images of frustule crystal structure analysis of *M. nummuloides* after organic matter removal at different time, frequencies and vacuum application

XRD analysis showed that organic matter was almost entirely removed using 4.5% NaOCl compared to the high temperature baked *M. nummuloide* (Fig. 4).

**Figure 5:**
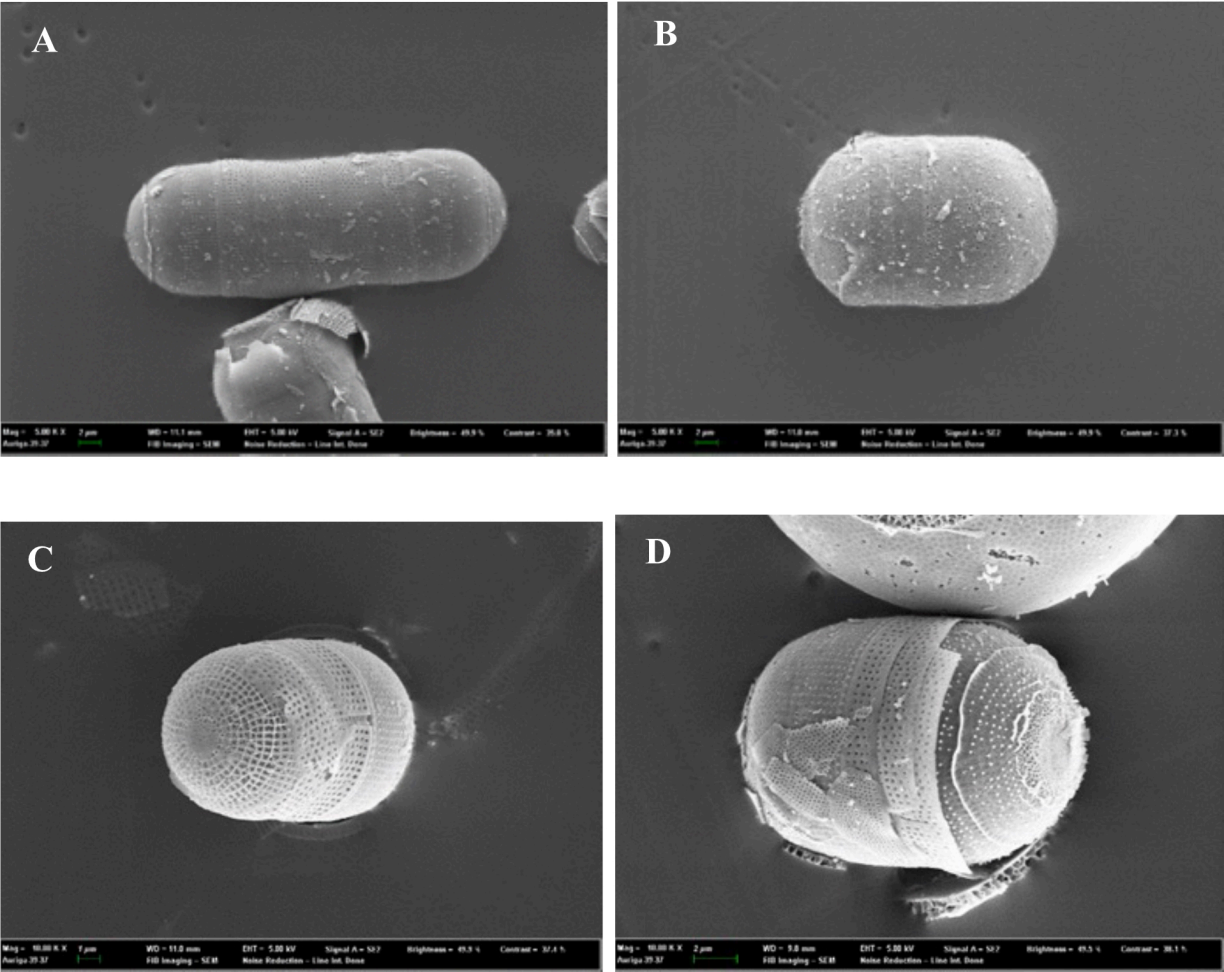
EDS images of *M. nummuloides* after organic matter removal A. Negative control: RM (Raw-material *Melosira*) B. Positive control: High temperature baked *Melosira* C. 170kHz, 60min, vacuum+ *Melosira* after organic matter removal D. 35kHz, 15min, vacuum− *Melosira* after organic matter removal

In the EDS analysis, the weight percent(wt.%) of the carbon(C), silicon(Si), and oxygen(O) elements showed no statistically significant differences based on vacuum application or stirring time. However, at 35kHz, significant differences were observed: the weight percent for the C element was 45.914 ± 8.635, and for the Si element, it was 20.545 ± 5.216. The p-value were 0.024 for C and 0.012 for Si, both below, signifying statistically significant differences. These findings are in close agreement with those of the positive control group, high temperature baked ‘melosira’, which showed a C weight percent of 44.65 and a Si element percent of 20.19 (Table 2, Fig. 5).

## Discussion

There are a variety of methods used to maintain hygiene and disinfect polymethyl methacrylate(PMMA) dentures. These methods can range from mechanical disinfection(manual scrubbing with toothpaste or soap) to chemical disinfection(soaking in disinfectant solutions) and physicochemical disinfection(using UV radiation and ultrasound), or a combination of these methods.^18^

Previous studies utilizing scanning electron microscopy or disclosing agents were limited due to their in vitro nature.^19–21^ Ultrasonic cleansing in an amphoteric surfactant solution was found to be highly effective.^22^ Interestingly, it was shown that weekly professional mechanical cleansing of the oral cavity, which included ultrasonic irrigation of dentures with a denture cleanser, significantly decreased the number of various oral bacterial strains compared to daily chemical mouth disinfection. This approach also reduced the incidence of aspiration pneumonia among the dependent elderly.^23^

Any remaining biofilm clusters can be removed by using cavitation at a higher acoustic pressure. Bacterial biofilm is rapidly disrupted by cavitation bubbles. Cleansing methods utilizing ultrasonic cavitation can be optimized by ensuring maximum contact between the cavitation bubbles and the surface. The results will contribute to more efficient ultrasonic cleansing of biofilms, and the protocol developed in this study can be applied to other research investigating surface cleansing.^24^

Existing methods for collecting frustules from diatom cells include oxidant cleansing, baking, and oxygen plasma etching. Oxidants commonly used to remove organic matter that covers or is within the silica structure include sulfuric acid^25,26^, hydrogen peroxide^27–29^, nitric acid^30^, or sodium dodecyl sulfate ethylenediaminetetraacetic acid solutions.^31^ Cleansing usually involves centrifugation, dilution, or filtration to remove oxidants and yield clean frustules. Baking at 400–800°C can also remove organic matter from diatom frustules.^32^ Furthermore, reaction products(carbon) may adsorb onto the frustules, becoming difficult to clean. Traditionally, concentrated sulfuric acid, known for its high efficiency, is used to clean frustules for 5–10 minutes at 60°C.

Ultrasonic cleansing leverages the cavitation effect and particle acceleration associated with ultrasound. Depending on the ultrasound and oscillator settings, various cavitation effects can be achieved, which means that the application of ultrasonic technology varies based on the material and structure of the object as well as the type and size of the particles to be removed. For particles larger than 1μm, frequencies of 38kHz, 100kHz, and 160kHz have shown efficiencies over 90%. Frequencies greater than 100kHz were necessary to remove particles smaller than 0.5μm. This study demonstrated particle removal efficiency using a cleansing device featuring vacuum ultrasonic technology, microbubble application technology, and vacuum drying technology. Additionally, by using electrolyzed ion water as an alternative cleansing agent, we achieved particle removal efficiencies of 99.55% for 1μm particles, 99.64% for 5μm particles, and 99.71% for 10μm particles.^33^

Despite numerous attempts under various conditions, no experimental variable demonstrated markedly distinct results. However, a general trend was observed: carbon was effectively removed at low frequencies, while nitrogen was more efficiently removed at high frequencies. This indicates that the physical force from cavitation plays a significant role in removing organic matter. In contrast, nitrogen appears to be eliminated by dissolving into the solution, particularly in the form of NaOCl. In such scenarios, it is the activation of the solution by agitation or vibration, rather than the intensity of cavitation, that becomes critical, with higher frequencies tending to offer an advantage. Notably, studies in wastewater treatment often use ultrasonic ranges of 300–500kHz. The organic matter removal in our experiment, while slightly inferior to that of the high-temperature baking positive control group, was still comparable. One possible explanation is that high-temperature baking increases diatom crystallinity, which prevents organic matter from re-adhering to the diatom surface. However, mechanical and chemical treatments using VAUS^™^ maintain an amorphous state, suggesting that the removed organic matter might reattach due to electrostatic forces. To address these limitations, it is postulated that employing plasma treatment could enhance organic matter removal rates.

In terms of nitrogen removal, many samples exhibited a complete removal rate of up to 100%, outforming the positive control group treated with high-temperature baking. The removal efficiency generally improved with increased frequency, longer stirrer times, and the application of a vacuum. Statistically, higher frequencies significantly correlated with greater removal efficiency. Among various organic matter removal solutions, including ferrous iron (Fe(II)), peracetic acid(PAA), the Fenton process, and sono-Fenton, the application of 4.5% NaOCl proved most effective. Coupling 4.5% NaOCl with mechanical stirring, ultrasonic application, and periodic negative pressure seemed to enhance organic matter removal from even the fine powder particles and the frustule pore structures.

XRD analysis, providing insights into crystalline structure, indicated that the 2 theta diffraction angle range (13 to 30 degrees) was characteristics of amorphous silica, as evidenced by the absence of distinct peaks and the broadness of the pattern.

EDX analysis, which visualizes elemental distribution on the sample surface, showed a decrease in carbon and an increase in silicon relative to the original melosira. While the removal rate did not match the high-temperature baked positive control group, it was nearly equivalent.

The use of sodium hypochlorite(NaOCl), commonly employed for dental root canal irrigation, resulted in the highest organic matter removal efficiency when compared to the Fenton-type process using peracetic acid(PAA) and the traditional Fenton process with ferrous iron and hydrogen peroxide. The removal efficiency was found to vary with the frequency and material used, with longer stirring times and the application of a vacuum being correlated with improved removal efficiencies. The VAUS^™^ developed in this study is anticipated to offer an environmentally friendly solution for producing high-quality amorphous bio-silica. It removes organic matters more quickly than traditional combustion or high-temperature baking methods. This system has enabled mechanical cleansing, which was previously impossible with conventional dental cleansing agents, marking a significant advancement-especially for the elderly with mobility issues, potentially enhancing their overall oral health.

Given the current shortage of effective chemical cleansing methods for biofilm eradication, the clinical application of VAUS^™^ could lead to transformative outcomes for oral and systemic health. Additionally, VAUS^™^ not only shortens the time needed to produce bio-silica but also significantly reduces the potential release of various toxic substances, addressing a major concern associated with traditional high-temperature baking methods. The integration of a magnetic stirrer in this innovative device indicates its broad applicability for processing smaller, lightweight particle materials in various ways compared to current equipment. With extensive research into four different frequencies at the prototype stage, determining the optimal conditions for specific applications will undoubtedly increase its utility in the future. The device’s compact and low-noise design makes it suitable for use in confined spaces and for individual research. Moreover, the reduced processing time suggests that VAUS^™^ can be a safe and rapid alternative for mass production when needed.

## Conclusions

Based on the experimental conditions of our study, the new procedure of applying periodic negative pressure during NaOCl pre-treatment has proven to be an effective method for chemically removing organic matter from diatoms to produce diatomic bio-silica. VAUS^™^ could be a valuable device for effectively removing organic matter from diatoms. With periodic vacuum ultrasonification and the aid of a magnetic stirrer, this experiment demonstrated the capability to remove organic matter comparably to the existing high-temperature baking process, but in a shorter time and using environmentally friendly chemical agents. This method holds promise for synthesizing high-quality amorphous bio-silica suitable for various applications, addressing the limitations of chemical treatments that use high-concentration toxic solution-known for their inferior ability to remove organic matter-and heat treatments that can increase crystallinity.

## Figures and Tables

**Table 1:** Measured values of total organic carbon(TOC) and total nitrogen(TN) RE(%) analysis

TOC RE(%)	Experimental group		Mean ± SD	*P* value

	Vacuum application	+	81.528 ± 3.276	0.599*

		-	82.081 ± 3.930	

	Sterring time (min)	15	80.929 ± 3.410	0.183**

		30	81.347 ± 3.874	

		60	83.138 ± 3.287	

	Frequency(kHz)	35	83.419 ± 3.052	0.008**

		72	82.870 ± 2.148	

		100	81.941 ± 3.233	

		170	78.989 ± 4.234	

TN RE(%)	Experimental group		Mean ± SD	*P* value

	Vacuum application	+	97.988 ± 4.262	0.904*

		-	97.840 ± 4.120	

	Sterring time (min)	15	98.388 ± 1.924	0.183**

		30	97.248 ± 4.971	

		60	98.106 ± 4.972	

	Frequency (kHz)	35	97.769 ± 5.916	0.757**

		72	98.168 ± 3.024	

		100	96.943 ± 5.021	

		170	98.775 ± 1.482	

* T-test,

** one-way ANOVA

**Table 2:** Measured values of C,Si,O mass norm(%) in EDS analysis

C	Experimental group		Mean ± SD	*P* value

Mass norm.(%)	Vacuum application	+	48.997 ± 9.559	0.313*

		-	51.415 ± 6.556	

	Sterring time (min)	15	51.120 ± 9.177	0.699**

		30	47.721 ± 8.008	

		60	51.776 ± 7.228	

	Frequency (kHz)	35	45.914 ± 8.635	0.024**

		72	47.571 ± 8.134	

		100	52.891 ± 6.766	

		170	54.447 ± 6.757	

Si	Experimental group		Mean ± SD	*P* value

Mass norm.(%)	Vacuum application	+	16.980 ± 6.406	0.733*

		-	16.450 ± 4.037	

	Sterring time (min)	15	16.971 ± 5.785	0.729**

		30	17.363 ± 5.570	

		60	15.811 ± 4.717	

	Frequency (kHz)	35	20.545 ± 5.216	0.012**

		72	17.002 ± 5.831	

		100	15.298 ± 3.833	

		170	14.017 ± 4.248	

O	Experimental group		Mean ± SD	*P* value

Mass norm.(%)	Vacuum application	+	34.112 ± 6.916	0.200*

		-	32.135 ± 2.747	

	Sterrring time (min)	15	31.908 ± 3.597	0.331**

		30	35.051 ± 7.843	

		60	32.410 ± 2.738	

	Frequency (kHz)	35	33.556 ± 3.506	0.214**

		72	35.594 ± 8.909	

		100	31.808 ± 3.020	

		170	31.535 ± 2.783	

* T-test,

** one-way ANOVA

## Data Availability

The datasets used and/or analysed during the current study are available from the corresponding author on reasonable request.
